# Supplementary material for: Lignin Accumulation in Three Pumelo Cultivars in Association with Sucrose and Energy Depletion
Source: Biomolecules. 2019 Nov 5;9(11):701. doi: 10.3390/biom9110701 (PMC6920757; doi:10.3390/biom9110701)
Supplement: Supplementary file 1 [file biomolecules-09-00701-s001.pdf]

Table S1 Correlationship between ATP, sugars and related enzymes with lignin in pericarp of BR.

Independent variables	Dependent variable (Lignin)	
	r	P
ATP	-0.987	0.01
Sucrose	-0.976	0.01
Total soluble sugar (0-30d)	0.93	0.05
Glucose	0.878	0.05
Fructose	0.822	0.05
PAL	0.911	0.05
PPO	0.977	0.01
POD	0.942	0.01

Table S2 Correlationship between invertases with sucrose in pericarp of BR.

Independent variables	Dependent variable (Sucrose)	
	r	P
NI	-0.93	0.05
S-AI	-0.925	0.05
B-AI	-0.984	0.01
